# Neuro-Behçet’s Disease With Coma

**DOI:** 10.7759/cureus.31596

**Published:** 2022-11-16

**Authors:** Mohamed Hamid, Soukaina Cherradi, Maha Ait Berri, Ahmed Bourazza

**Affiliations:** 1 Neurology, Mohamed V Military Teaching Hospital, Mohamed V University, Rabat, MAR; 2 Obstetrics and Gynaecology, Ibn Sina Hospital, Mohamed V University, Rabat, MAR; 3 Neurology, Moulay Ismail Military Hospital, Meknes, Sidi Mohamed Ben Abdellah University, Fez, MAR

**Keywords:** brain stem, neuro-behçet’s disease, coma, cyclophosphamide, neuroradiology

## Abstract

Behçet’s disease (BD) is a multisystemic vasculitis condition with unknown pathophysiology. Its clinical manifestations include recurrent oral and genital ulcers, cutaneous lesions, and uveitis. The term neuro-Behçet’s disease (NBD) refers to the predominant neurological involvement in BD. The management of NBD is based on the severity of symptoms and the existence of additional systemic signs. Glucocorticoids and disease-modifying drugs are used for the treatment of the disease. In this report, we present a case of neuro-Behçet’s disease (NBD) that presented with coma and extensive neuroradiological abnormalities.

## Introduction

Behçet’s disease (BD) is an autoimmune vasculitis characterized by a combination of cutaneous lesions, uveitis, genital ulcers, and recurrent oral ulcers. Less than 10% of patients with BD present neurological symptoms. The involvement of the nervous system in BD constitutes the diagnosis of neuro-Behçet’s disease (NBD) [1]. The correlation between clinical signs, neuroradiology, and cerebrospinal fluid analysis is necessary to diagnose NBD.

The management of NBD depends on the existence of additional systemic signs and the severity of the neurological symptoms. Glucocorticoids and disease-modifying medication are frequently used as the first line of treatment for NBD [2]. We describe a rare case of neuro-Behçet’s disease (NBD) presenting with coma and extensive neuroradiological abnormalities.

## Case presentation

A 35-year-old male with a history of recurrent oral ulcers, genital aphthosis, and panuveitis was admitted to the emergency department with an acute loss of consciousness. The patient received azathioprine and cyclosporine four years ago for BD. After that, the patient had been out of sight for three years prior to his admission to the hospital.

**Figure 1:**
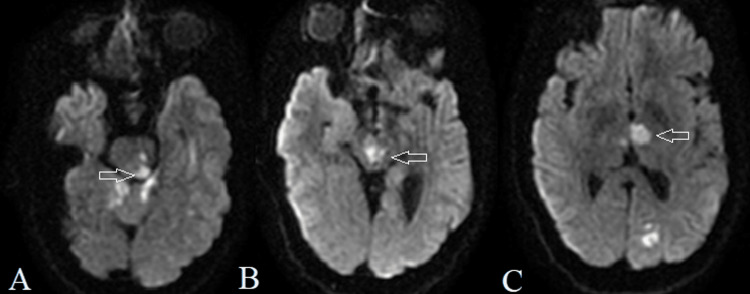
Cranial magnetic resonance DWI showing hyperintense signal at pontine tegmentum extending to the midbrain (A and B), thalami, and left occipital lobe (C) (arrows). DWI: diffusion-weighted imaging

**Figure 2:**
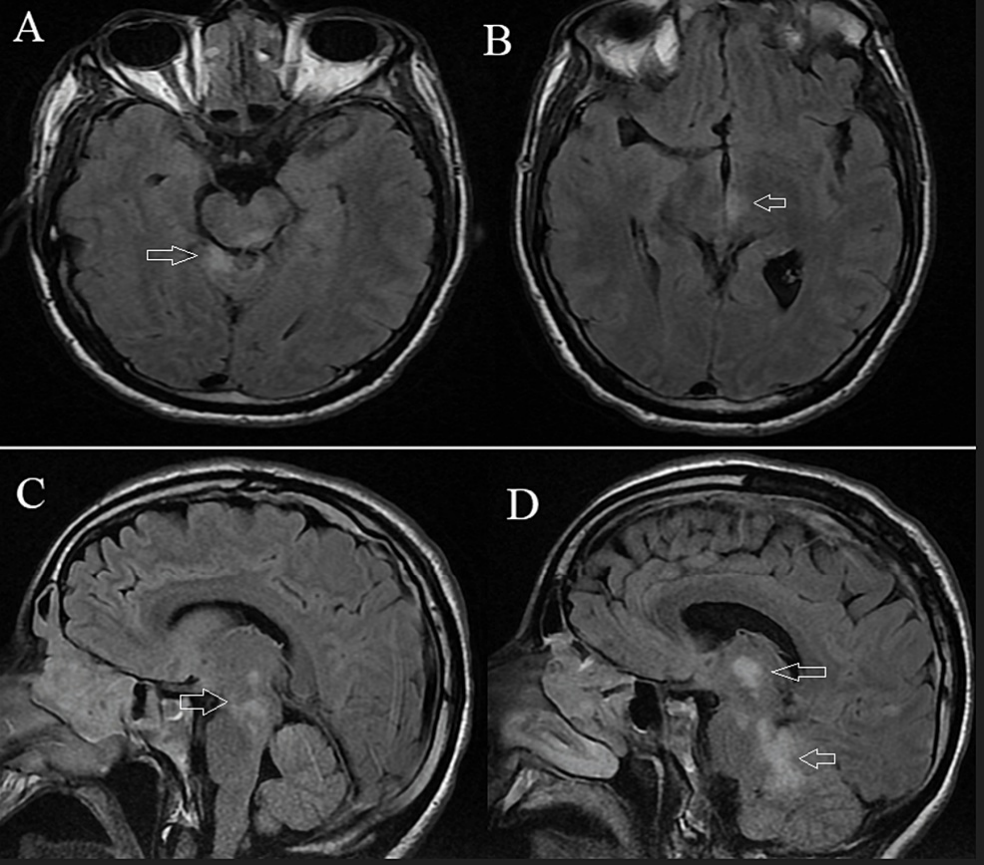
Cerebral magnetic resonance imaging on axial fluid-attenuated inversion recovery sequence demonstrating lesion in the cerebellar vermis (A) and left thalamus (B) (arrows). The sagittal section identifies extensive lesions of the meso-diencephalic region (C and D) (arrows).

A neurological evaluation showed an unconscious patient with a Glasgow Coma Scale score of 9/15 (eye opening: 2; verbal response: 3; motor response: 4). He had a supple neck with positive oculocephalic reflexes. Deep tendon reflexes were brisk with a bilateral Babinski sign. The examination of the other systems was intact. Large paraclinical tests were normal (hemogram, C-reactive protein (CRP) test, B12 and B9 seric levels, thyroid hormone, serum electrolytes, and renal and hepatic tests). Cerebral magnetic resonance imaging demonstrated extensive hyperintensity lesions widening from the tegmental region of the pons to the midbrain, cerebellar vermis, thalami, and left occipital lobe (Figure 1 and Figure 2).

**Figure 3:**
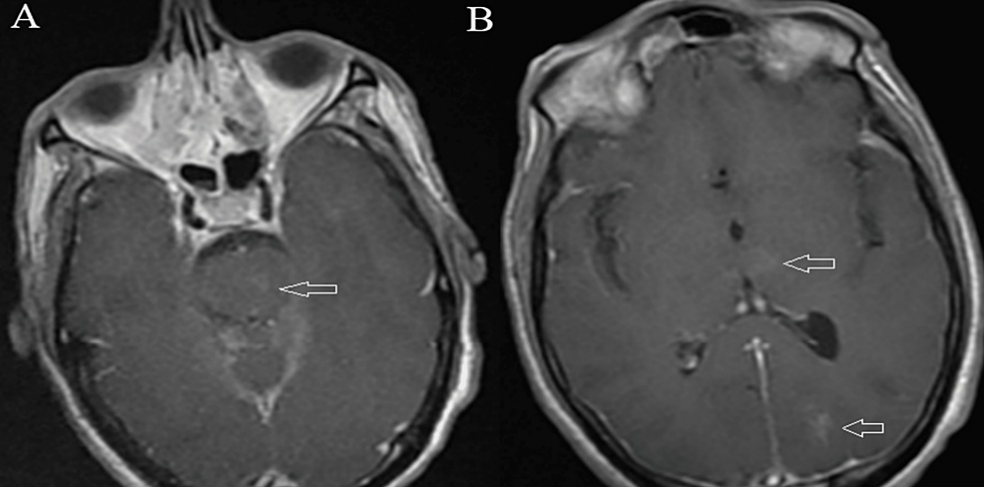
Cranial magnetic resonance imaging on contrast-enhanced T1-weighted images showing small areas of enhancement in the pons (A) (arrow), thalami, and left occipital region (B) (arrows).

Cerebral magnetic resonance imaging on contrast-enhanced T1-weighted images identified areas of enhancement in the pons, thalami, and left occipital region (Figure 3). These findings suggested that the patient has active inflammatory lesions of the central nervous system.

The cytochemical study of cerebrospinal fluid was normal. The autoimmune and human immunodeficiency virus and angiotensin-converting enzyme tests were negative. Computed tomography of the chest, abdomen, and pelvis was normal. The human leukocyte antigen type was B51.

The patient received treatment with an intravenous methylprednisolone bolus (1 g/day for five days) and then 1 g/day once a week for four weeks and intravenous cyclophosphamide (1 g/month). A 60 mg/day oral steroid tapering dosage was administered, with eight weeks of gradual degression up to a minimum dose of 5 mg/day. Evolution was favorable, with the recovery of consciousness after one week of his admission. The patient had a mild residual left hemiparesis with no complications or systemic recurrence over the six-month follow-up period.

## Discussion

The first description of neurological signs as a part of BD dates back to 1941. In 1954, an Italian ophthalmologist used the term “neuro-Behçet” [3]. Although the fundamental cause of BD is unclear, it is thought that autoimmune vasculitis is the source of its clinical symptoms. NBD is developed through the same mechanism as other systemic BD symptoms. Different regions of the central nervous system could be affected by NBD, such as the brain stem, basal ganglia, thalamus, cerebellum, and internal capsule [4].

The human leukocyte antigen B51 is considered the main genetic marker linked to BD [5]. Other causes of BD are the aberrant activation of neutrophils and the implication of the vascular endothelium [6]. Generally, neurological symptoms will appear five years after the onset of non-neurological symptoms in BD [1]. The most common clinical symptoms of NBD include motor dysfunction, cranial nerve palsy, and cognitive impairment. Neurological deficits are often consistent with lesions detected on magnetic resonance imaging, which frequently extend from the brain stem to the deep grey nuclei. On T2-weighted images, acute lesions appear as hyperintense foci that enhance with contrast [7].

Our patient presented with a state of unarousable unconsciousness. Coma is caused by structural brain stem lesions, including the ascending reticular activating system. Anatomically, this arousal system is constituted by various structures in the rostral brain stem tegmentum and the diencephalon [8]. In our case, the parenchymal lesions were extensive from the dorsal pons to the thalami passing via the ascending reticular activating system.

To prevent lasting organ damage, the main objective of treatment for BD is to decrease inflammatory flare-ups and limit recurrences. The treatment of BD includes corticosteroids and colchicine, while immunomodulators are recommended for more severe diseases. NBD necessitates a distinct therapeutic approach. The first-line agents are azathioprine, mycophenolate, and methotrexate. For severe parenchymal lesions and encephalitis, high-dose corticosteroids, cyclophosphamide, and tumor necrosis factor-alpha inhibitor must be used [9]. Generally, NBD is not related to a good prognosis. Although neurological disease activity decreases over time, cumulative damage increases since neurons cannot recover [10].

## Conclusions

BD is an autoimmune vasculitis disorder that can affect the nervous system. Our clinical case shows that a flare-up of NBD can occur through a deep coma corresponding to extensive brain stem and diencephalon lesions. Clinicians should consider NBD as a possible cause of acute loss of consciousness. An accurate diagnosis of the disease will allow the initiation of prompt treatment with corticosteroids and immunomodulators, in the hopes of a favorable outcome. To further understand the various potential mechanisms that cause coma in NBD, more studies are needed.
